# Recent advances in the application of artificial intelligence and wearable devices in volleyball

**DOI:** 10.3389/fspor.2026.1855108

**Published:** 2026-08-05

**Authors:** Guobao Zhou, Xinlong Shi, Yidong Wang, Kaixin Xu, Weiquan Shi, Qinglei Li, Jaeyoung Park

**Affiliations:** 1 College of Art and Physical Education, Kyungil University, Gyeongsan-si, Gyeongbuk-do, Republic of Korea; 2 Graduate School, Daegu University, Gyeongsan-si, Gyeongsangbuk-do, Republic of Korea

**Keywords:** deep learning, machine learning, training and match applications, volleyball, wearable devices

## Abstract

Volleyball is characterized by high intensity, rapid pace, and multi-agent coordination, where performance depends not only on individual technical execution but also on collective behavior organization, offense–defense transition efficiency, and dynamic decision-making. Conventional volleyball performance analysis, which relies on expert judgment, manual statistics, and video replay, is constrained by labor-intensive analytical workflows and delayed feedback, limiting its ability to continuously and accurately capture complex behaviors during training and competition in real time. Moreover, the analytical outcomes cannot be translated promptly into targeted training interventions and evidence-based decision support. In recent years, advances in artificial intelligence and wearable technologies have emerged as promising solutions to these limitations, accelerating the transition of volleyball performance analysis from an experience-driven to a data-driven paradigm. This review systematically summarizes research progress in this field. It first examines the applications of machine learning and deep learning in motion recognition, event detection, behavior modeling, and outcome prediction. It then summarizes data acquisition foundations based on inertial measurement units, flexible sensors, and multimodal integration, and further synthesizes their applications in match and training analysis, athlete performance quantification and load assessment, training decision-making and tactical optimization, and match outcome prediction. Finally, the key challenges in current research are discussed, and future directions for the intelligent development of volleyball are outlined, providing a systematic reference for advancing intelligent volleyball analytics.

## 1 Introduction

Volleyball training and competition are not a mere aggregation of isolated technical actions; rather, they constitute a dynamic system jointly shaped by individual motor execution, collective coordination, and continuous offensive–defensive transitions (1–3). Both key technical skills—such as spiking, blocking, serve reception, and jumping—and collective behaviors—including formation organization, rhythm modulation, and in-game adaptation—are inherently dependent on sustained interactions across multiple temporal scales (4–7). Therefore, the core of volleyball performance analysis lies not simply in identifying the completion of a specific action, but in accurately characterizing the coupling relationships among movements, events, workload, and decision-making within the continuously evolving processes of training and competition. For a long time, volleyball training and match analysis have predominantly relied on video replay, manual statistics, and coaching experience–based judgment (8–10). For example, continuous monitoring of athletes’ jump frequency and jump height enables quantitative assessment of training load and fatigue status, allowing coaches to identify excessive workload, optimize training intensity, recovery protocols, and player rotation strategies in a timely manner, and ultimately support evidence-based training decisions and injury risk management (11). Although these approaches provide practical value for technical review and experiential knowledge accumulation, they are largely retrospective in nature. Consequently, they remain limited in their ability to continuously, precisely, and in real time capture critical events during high-speed offensive–defensive transitions, as well as dynamic variations in athlete workload and patterns of team coordination.

Particularly in high-level volleyball contexts, the game is characterized by rapid tempo, complex interaction patterns, and clearly defined positional roles. Under such conditions, conventional approaches often struggle to simultaneously address action recognition, state assessment, and decision support, resulting in a noticeable disconnect among training intervention, tactical adjustment, and game understanding (9, 12, 13). Against this backdrop, volleyball analytics has progressively transitioned from experience-driven paradigms toward data-driven frameworks, thereby accelerating the adoption of artificial intelligence (AI)–based methodologies within the field. In recent years, advances in machine learning (14–16) and deep learning (17, 18) have opened new analytical pathways for action recognition, event extraction, behavior modeling, and outcome prediction in volleyball. Concurrently, developments in wearable sensing technologies (19–21), computer vision (22–24), and multimodal data acquisition (25, 26) have enabled the continuous recording and quantitative characterization of athletes’ movements, trajectories, workload, and certain physiological states. With the ongoing integration of these techniques into sports analytics, related research has evolved beyond single-action detection or isolated outcome analysis, extending toward comprehensive applications encompassing match and training analysis, athlete performance quantification, training decision support, tactical optimization, as well as game prediction and virtual simulation. For example, integrating multimodal information, including players’ on-court positions, movement trajectories, jump kinematics, and technical actions, enables the identification of offensive–defensive transitions, tactical execution, and movement quality, thereby facilitating the development of targeted training interventions and tactical optimization strategies (27). Collectively, these developments indicate that the role of AI in volleyball is shifting from a localized recognition tool to an integrated technological framework supporting complex performance analysis and decision-making processes (23, 28, 29).

Despite the growing body of related research, systematic investigations into how AI methods and wearable sensing technologies can be effectively deployed in real-game scenarios to support analysis and decision-making in training and competition remain limited. In this context, this study provides a comprehensive review of the application of AI and wearable devices in volleyball. First, the principal methodological frameworks of machine learning and deep learning for volleyball performance analysis are outlined. Second, the data acquisition foundations based on inertial measurement units (IMUs), flexible sensors, and multimodal sensing systems in volleyball are summarized. Building upon this, the representative applications of these approaches are further synthesized, including match and training analysis, athlete performance quantification and workload assessment, training decision support and tactical optimization, as well as match outcome prediction and virtual simulation. Finally, future directions for intelligent volleyball analytics are discussed from the perspectives of multimodal data fusion, adaptive learning, and closed-loop intelligent decision-making, with the aim of providing insights for scientific training, match analysis, and decision support in volleyball.

## 2 Artificial intelligence in volleyball

With the rapid development of data acquisition, computational modeling, and intelligent decision-making technologies, AI has become an important methodological foundation for volleyball analytics. Existing studies have mainly adopted machine learning and deep learning models to address tasks such as match outcome prediction, athlete performance evaluation, key factor identification, action recognition, event detection, and multi-agent behavior understanding. These models differ substantially in terms of input data types, target tasks, interpretability, modeling capability, and practical limitations. Therefore, before discussing specific methodological categories, the representative AI models used in volleyball analysis, together with their input data, major tasks, advantages, and limitations, are summarized to provide an overall methodological framework for this section (see Table 1).

**Table 1:** Characteristics, advantages, and limitations of representative AI models for volleyball performance analysis.

AI category	Representative model	Input data	Primary task	Advantages	Limitations	Refs.
Machine learning	Regression and discriminant models	Match statistics and technical performance indicators	Match outcome prediction, player performance evaluation, and identification of key performance determinants	High interpretability; suitable for analyzing variable relationships and identifying influential performance factors	Limited capability for modeling nonlinear relationships and complex spatiotemporal patterns	(30)
		Technical performance indicators, match-phase indicators, and match outcomes	Match outcome prediction, identification of key performance indicators, and player performance evaluation	Highly interpretable; enables quantitative assessment of the contribution of individual technical indicators and identification of key performance determinants	Relies on manually designed features; limited in capturing complex nonlinear relationships and temporal dependencies	(13)
		FMS, SEBT, CMJ height, agility *T*-test, body height, BMI, etc.	Sports injury risk prediction and identification of high-risk athletes	Simple model structure with high interpretability; quantifies the contribution of individual indicators to injury risk and provides risk thresholds	Depends on handcrafted features; mainly suitable for linear relationships and incapable of learning complex nonlinear and temporal patterns	(31)
	Support vector machine	Acceleration and angular velocity signals, with temporal features extracted after PCA-based dimensionality reduction	Volleyball action recognition and classification	Strong performance on small-sample datasets; high classification accuracy and good generalization capability	Relies on feature engineering and parameter tuning; computational efficiency decreases with large-scale datasets	(32)
		Visual features extracted from volleyball images	Spike (release) angle recognition and action correction	Good generalization ability; suitable for small-sample nonlinear classification and accurate action recognition	Relies on handcrafted feature extraction and parameter optimization; limited feature representation capability in complex scenarios	(33)
	Decision trees	Anthropometric characteristics and technical performance statistics	Match outcome prediction and identification of key determinants of victory	Capable of modeling complex nonlinear relationships, automatically evaluating feature importance, and providing good interpretability	Sensitive to hyperparameter settings; relatively high training cost; limited capability for modeling temporal dependencies	(34)
		Training load, jump load, health status, and match performance data from multiple sources	Match performance prediction and identification of key performance determinants	Capable of handling high-dimensional heterogeneous data, evaluating feature importance, and modeling complex nonlinear relationships	Requires relatively large sample sizes; limited interpretability; difficult to capture temporal dynamics	(35)
	Probability and temporal models	Video data (RGB images, optical flow, and temporal features)	Volleyball action recognition and behavior analysis	Capable of modeling temporal dependencies in action sequences; suitable for sequential behavior recognition; good temporal modeling capability	Limited representation of high-dimensional features; relies on handcrafted feature extraction; difficult to capture long-range temporal dependencies	(36)
		Match event data (serves, receptions, sets, spikes, blocks, defensive actions, etc.)	Individual player contribution evaluation, point-winning probability estimation, and match performance analysis	Capable of modeling rally evolution, quantifying the contribution of different technical actions to point-winning probability, and providing a unified framework for player performance evaluation	Relies on high-quality sequential event annotations; model construction is relatively complex and computationally expensive	(37)
Deep learning	Detection and tracking networks	Volleyball match video data	Player detection and localization	High detection accuracy and localization precision; suitable for real-time multi-player detection and provides reliable targets for subsequent tracking and action recognition	Susceptible to target occlusion, scale variation, and complex backgrounds; long-term tracking usually requires temporal modeling	(38)
		Volleyball match videos	Volleyball detection and trajectory estimation	High detection accuracy and localization precision; robust to multi-scale targets and suitable for real-time object detection	Detection performance for small targets and complex scenes remains affected by occlusion and background interference; typically requires post-processing or tracking algorithms for improved performance	(39)
	Convolutional neural networks	Human pose keypoints and trajectory data extracted from volleyball videos	Group activity recognition and individual action classification	Effectively captures spatial–temporal features with good generalization capability while maintaining high recognition accuracy and computational efficiency	Relies on accurate pose estimation and object tracking; recognition performance in complex scenes may be affected by pose estimation errors	(40)
		Volleyball action videos (RGB frames, grayscale images, and fused Crop-region features)	Volleyball action recognition	Simultaneously captures spatial–temporal features, improves action recognition accuracy, and reduces model complexity	Requires large-scale video and training datasets; model generalization capability still requires further validation	(41)
	Recurrent neural networks	Volleyball action videos (continuous video frames)	Volleyball action recognition	Effectively captures temporal dependencies in action sequences, reducing computational complexity while improving recognition accuracy and real-time performance	Limited capability for modeling long-range temporal dependencies and complex scenarios; performance relies on high-quality video data	(42)
		Volleyball action videos and human skeletal keypoint data	Volleyball posture recognition and action analysis	Effectively models temporal variations and inter-joint spatial relationships, improving posture recognition accuracy and robustness	Relies on accurate pose estimation; relatively complex network architecture and high computational cost	(43)
	Transformer	Volleyball match videos	Group activity recognition and player localization	Leverages hierarchical query representations and distributed attention mechanisms to model spatiotemporal dependencies, enabling simultaneous recognition of individual and group activities with high accuracy	Complex model architecture; computationally intensive; high training cost for large-scale Transformer models	(44)
		Three-dimensional human skeletal joint sequences	Action recognition and group activity recognition	Effectively models local joint motion, global action semantics, and human interaction relationships, enabling hierarchical learning of spatiotemporal action representations	Complex model architecture; computationally intensive; high processing cost for long-sequence training and multi-person data	(45)
	Graph neural networks	Volleyball match videos	Group activity recognition	Effectively models multi-person interactions and action semantics, enabling joint reasoning over player relationships and scene information while improving group activity recognition accuracy	Jointly models local player interactions and global scene relationships, effectively capturing spatial dependencies and interaction information to improve group activity recognition accuracy	(46)
		Volleyball match videos	Group activity recognition	Jointly models local player interactions and global scene relationships, effectively capturing spatial dependencies and interaction information to improve group activity recognition accuracy	Complex model architecture; relies on accurate object detection and localization; computationally intensive, particularly in large-scale scenarios	(47)

CMJ, countermovement jump; FMS, functional movement screen; SEBT, star excursion balance test; PCA, principal component analysis; RGB, Red–Green–Blue.

### 2.1 Machine learning

Machine learning refers to a class of data-driven computational approaches that learn the mapping between inputs and outputs, enabling the extraction of latent patterns from historical data without explicitly defined rules, and thereby supporting classification, prediction, and decision-making tasks (48, 49). In volleyball, with the continuous improvement of match data acquisition and statistical methodologies, research has gradually shifted from traditional experience-based analysis and descriptive statistics toward task-oriented, data-driven modeling (6, 50). In this process, machine learning techniques have been widely applied to the analysis of technical performance indicators, physiological state parameters, and behavioral feature data (51). These approaches not only enable match outcome prediction and key factor identification, but also support athlete performance evaluation, injury risk analysis, and tactical decision optimization (52). At present, existing studies can be broadly categorized into four representative methodological groups. The first comprises regression and discriminative models, represented by logistic regression (53, 54) and linear regression (55), which emphasize the interpretability of variable relationships and the quantification of risk (56). The second includes kernel-based methods, typified by support vector machines (SVM) (57–59), which are well suited for nonlinear classification under small-sample conditions. The third category consists of ensemble learning models, represented by random forests (60–62), which exhibit strong performance in high-dimensional feature prediction and key variable selection (63). The fourth involves probabilistic temporal models, including hidden Markov models (HMM) (64) and Markov chains (65), which are used to characterize the dynamic evolution of actions and rally sequences (66). Overall, machine learning methods have promoted the transition of volleyball analysis from experience-driven approaches to quantitative modeling, and have established a methodological foundation for subsequent, more advanced intelligent analytics.

#### 2.1.1 Regression and discriminative models

**Figure 1:**
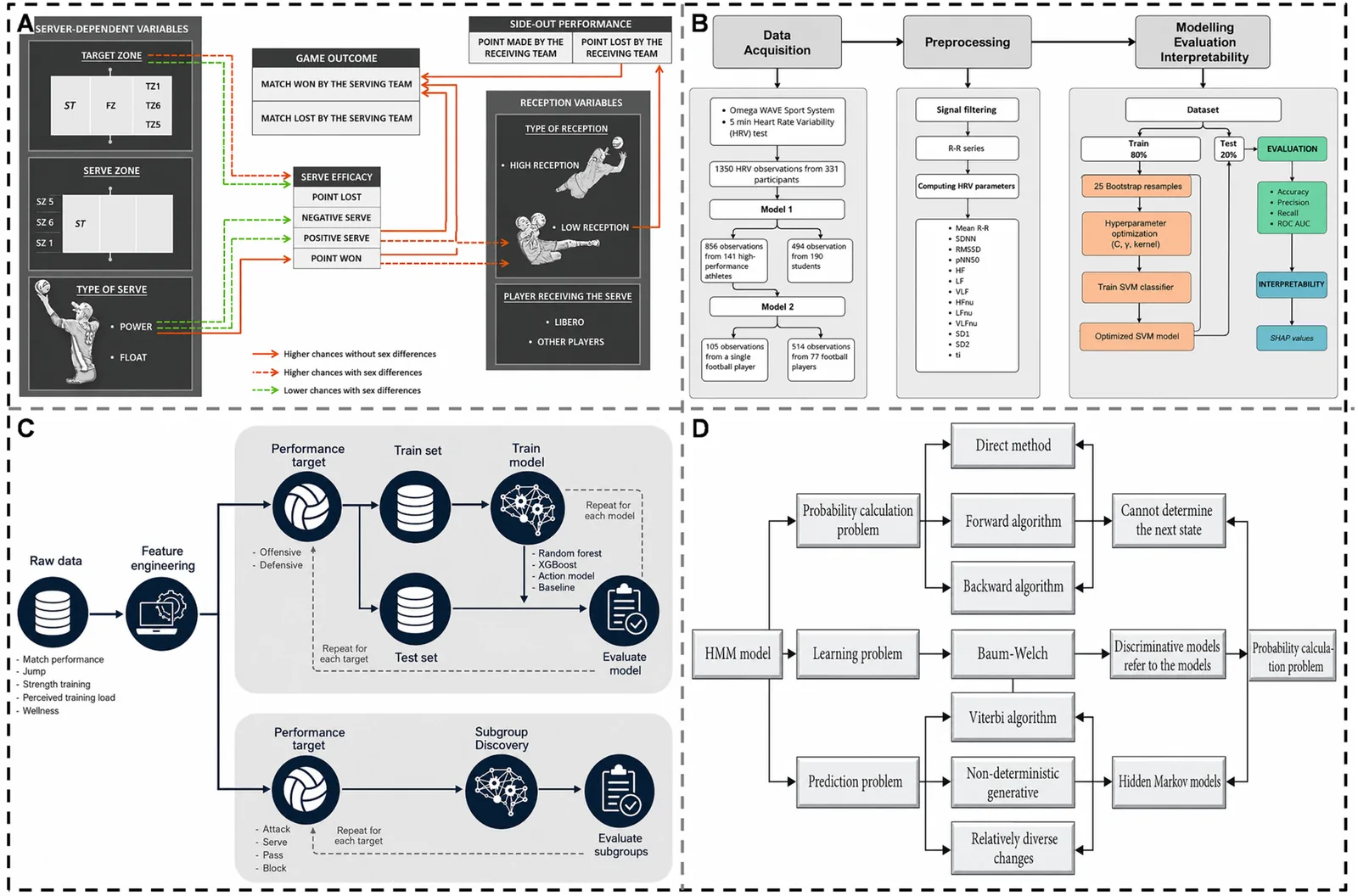
Machine learning-assisted analysis in volleyball. **(A)** Data-driven analysis of the serve–receive process in sitting volleyball (30). **(B)** Workflow of the SVM model, from data acquisition to interpretability (71). **(C)** Machine learning-based performance analysis in volleyball (35). **(D)** Schematic diagram of the HMM framework (36).

In the evolution of volleyball data analysis from descriptive statistics to model-driven approaches, regression and discriminative models represent one of the earliest and most widely adopted methodological frameworks (67). Parametric models, including logistic regression, linear regression, and their regularized variants (e.g., ridge regression and least absolute shrinkage and selection operator), enable a relatively direct characterization of the relationships among technical performance indicators, physiological states, and match outcomes. As such, they hold particular value in studies that emphasize interpretability and risk quantification. Cavedon et al. (30) demonstrated, using a mixed-effects logistic regression model, that serving does not influence match outcomes in isolation but rather operates through a chained pathway from serving strategy to final outcome (Figure 1A). Specifically, effective serving can induce low-quality receptions, significantly increasing the probability of side-out errors and thereby enhancing the likelihood of victory for the serving team. Their findings further indicate that serve placement exerts a significant effect on performance, with serves directed toward the deep central zone being more advantageous, whereas power serves, although more aggressive, are associated with a higher risk of error. Notably, da Costa et al. (13) established the relationship between technical indicators and set outcomes using a logistic regression model, achieving an accuracy of approximately 90.50% in win–loss prediction. This result further validates the applicability of such models in volleyball match outcome modeling.

Beyond match outcome analysis, regression and discriminative models are also widely used to assess individual athlete status and injury risk. Wang et al. (68) reported, based on logistic regression, that asymmetry in knee extensor concentric peak torque is a significant injury risk factor in volleyball [odds ratio (OR) = 1.64, 95% confidence interval (CI): 1.14–2.37, *p* < 0.01], while asymmetries in single-leg CMJ height (OR = 1.18) and *T*-test performance (OR = 1.41) are also significantly associated with injury risk. Erol et al. (31) further demonstrated that discriminative models based on functional movement and jumping metrics can effectively stratify injury risk, achieving an area under the curve (AUC) of 0.89. In addition, Carvalho de Toledo et al. (69) found through multivariate logistic regression that scapular dyskinesis is significantly associated with shoulder pain, with prior shoulder injury history exerting an even stronger effect. These models have also been applied to cognitive and decision-making analysis; for example, Ittlinger et al. (70) used linear regression to quantify the relationship between cognitive bias and win probability estimation error. Despite their advantages, these models are typically based on linear assumptions or predefined functional forms, limiting their ability to capture complex nonlinear patterns and high-dimensional feature interactions, which has driven the adoption of more advanced nonlinear methods.

#### 2.1.2 Support vector machines

To address the limitations of traditional parametric models in capturing complex nonlinear relationships, SVM and other kernel-based discriminative models have been introduced into volleyball data analysis (67). By mapping original features into a high-dimensional kernel space, these methods enable effective separation of nonlinear decision boundaries and demonstrate clear advantages in tasks involving small samples, high-dimensional features, and complex classification structures. In volleyball, SVM has been widely applied to action recognition, state classification, and performance evaluation. For example, Sun et al. (32) proposed a mobile inertial sensor-based method for volleyball stroke recognition. Motion data acquired by inertial sensors were first preprocessed through resampling and smoothing-based denoising, followed by feature extraction and classification model training. This workflow enabled the automatic recognition and classification of volleyball technical actions. The method achieved an accuracy of up to 96%, which could be further improved to 98% with parameter optimization. Building upon this, research has extended from observable movements to internal physiological states. Estrella et al. (71) proposed a heart rate variability (HRV)-based approach for athletic performance identification by collecting resting-state HRV data, extracting representative HRV features through signal preprocessing and feature extraction, and subsequently training an SVM model for classification (Figure 1B). Shapley additive explanations analysis was further employed to enhance model interpretability, enabling automatic identification of athlete status and quantitative evaluation of the contributions of key HRV features to model predictions, with the SVM model achieving an accuracy of 84% and an area under the receiver operating characteristic curve of 0.91. In parallel, SVM has also been introduced into injury risk management; de Leeuw et al. (72) applied SVM for injury monitoring in volleyball players, achieving fully individualized risk classification.

SVM also demonstrates strong adaptability in vision-driven volleyball scenarios. Fang et al. (33) employed kernel-based SVM to identify and correct athletes’ hitting angles, providing support for the parametric analysis of technical actions. In addition, by integrating visual images with skeletal keypoint features, the accuracy of erroneous action recognition can be improved from approximately 70% to over 95% (73). These findings indicate that SVM offers strong classification performance and robust applicability in volleyball action recognition and state classification. However, its performance is highly dependent on kernel selection and parameter tuning, and its interpretability is generally weaker than that of regression-based models. Moreover, computational complexity increases significantly with larger sample sizes. Consequently, for tasks emphasizing predictive stability, multivariate feature integration, and key factor selection, research has increasingly turned to ensemble learning approaches.

#### 2.1.3 Decision trees

In efforts to further enhance predictive performance and variable selection capability, ensemble learning methods based on decision trees have been widely adopted. Approaches such as random forests (74), gradient boosting decision trees (GBDT) (75), and XGBoost (76) integrate multiple weak learners to improve model stability, robustness, and generalization, thereby demonstrating clear advantages in volleyball data analysis tasks involving multivariate inputs and complex outputs. For example, de Leeuw et al. (35) developed a machine learning framework for volleyball performance analysis (Figure 1C). In this framework, multi-source data—including match performance, jumping ability, strength training, training load, and physiological condition—were used as inputs. Through feature engineering, statistical features, including the mean, quartiles, standard deviation, and sum, were aggregated over multiple temporal windows (7, 14, and 28 days) to construct predictive features, which were subsequently used to develop predictive models for offensive and defensive performance. Random forest and extreme gradient boosting models were subsequently applied to establish the mapping from input features to performance outcomes. The results demonstrated that the model achieved mean absolute errors (MAEs) of approximately 0.91 and 1.15 for offensive and defensive performance prediction, respectively. Compared with the baseline model, the prediction errors were reduced by approximately 46.8% and 59.4%, respectively, indicating high predictive accuracy and robust performance.

Building upon this, ensemble learning methods have been further extended to diverse task scenarios. For instance, Ming et al. (34) developed a match win probability prediction model based on GBDT, achieving a mean relative error of 5.30% and a coefficient of determination (*R*^2^) of 0.743, indicating good fitting performance. Similarly, Sanjaykumar et al. (77) developed a random forest regression model to predict volleyball players’ performance scores based on demographic characteristics, such as age and height, together with physical attributes, including body fat percentage, muscle mass, bone mass, and body mass index (BMI). The model achieved an *R*^2^ of 0.9418. Beyond prediction tasks, decision tree–based models also exhibit strong capability in identifying key influencing factors. Peng et al. (78), using a CART decision tree to analyze beach volleyball data, found that attack success rate is the primary determinant of match outcomes; when it exceeds 55%, the probability of winning increases significantly. Overall, ensemble learning methods offer clear advantages in predictive accuracy, multivariate data integration, and key feature selection, making them well suited to the complex, multi-source nature of volleyball data analysis. However, these methods are often associated with increased model complexity and reduced interpretability compared to traditional regression models, and they are less capable of explicitly capturing temporal dependencies in match processes. This limitation has led to the growing adoption of probabilistic and temporal models as complementary approaches for understanding action continuity and rally dynamics.

#### 2.1.4 Probabilistic and temporal models

Given the strong temporal dependencies in volleyball matches, most conventional models—being largely static—are insufficient for capturing the dynamic evolution of actions and rally sequences. Consequently, probabilistic and temporal models have gained increasing attention. Methods such as HMM and Markov chains describe dynamic relationships between consecutive actions and within rallies from a state transition perspective, offering advantages in behavior recognition and process modeling (79–81). For example, Wang et al. (36) proposed an HMM-based temporal modeling framework for sports video action recognition (Figure 1D). Video sequences are used as input, with spatiotemporal features extracted and state transitions modeled via HMM. The forward–backward algorithm is applied for state probability estimation, Baum–Welch for parameter learning, and Viterbi for optimal state decoding. This approach enables sequential modeling of actions such as serving, spiking, and movement, effectively capturing temporal dependencies. Experimental results show an accuracy of up to 92.7%, with over 86% accuracy for complex actions, demonstrating strong temporal modeling capability. Probabilistic transition mechanisms have also been used to quantify state evolution within rally processes. Powers et al. (37) developed a rally-level Markov chain model that quantified player contributions by tracking dynamic changes in point-winning probability throughout rally state transitions. Similarly, Chacoma et al. (12) proposed a stochastic process–based model of rally dynamics, using two probabilistic parameters to describe match behavior, and achieved a close fit to real match data (Jensen–Shannon divergence = 0.009). These studies demonstrate that probabilistic and temporal models are not only effective for continuous action recognition, but also provide a novel framework for analyzing state transitions and contribution evaluation in match processes. However, such models still rely heavily on manually designed features and are limited in handling high-dimensional unstructured data, such as video and image inputs. As volleyball data have increasingly evolved from structured statistics to video streams, skeletal sequences, and multimodal signals, research has consequently shifted toward models with stronger automatic representation learning capabilities.

### 2.2 Deep learning

Compared with traditional machine learning approaches that rely on structured data and handcrafted feature engineering, deep learning employs an end-to-end learning paradigm to automatically extract multi-level feature representations directly from raw data, such as videos, images, and sensor signals, thereby substantially reducing dependence on prior feature design (82, 83). From a modeling perspective, deep learning leverages nonlinear transformations within multilayer neural networks to achieve hierarchical representation learning, progressing from low-level perceptual features to high-level semantic representations, and demonstrating clear advantages in handling high-dimensional unstructured data and complex scenarios (84, 85). In volleyball analytics, its application has evolved from basic perception and spatial feature modeling toward temporal modeling, global dependency learning, and multi-agent interaction modeling (86). Overall, deep learning not only enhances the automation of action recognition and behavior analysis in volleyball, but also drives a shift from single-action detection to the understanding of collective coordination and tactical pattern modeling.

#### 2.2.1 Detection and tracking networks

In the early stages of applying deep learning to volleyball analysis, object detection and multi-object tracking networks provided critical support for structuring match data. For instance, object detection models based on the You Only Look Once (YOLO) series (87–89) enable automatic localization of players and the ball, while multi-object tracking algorithms further extract continuous trajectory information, forming the foundation for subsequent action recognition and behavior analysis. Accordingly, research at this stage has primarily focused on improving detection accuracy, trajectory continuity, and robustness in complex match scenarios. For example, Jiang et al. (38) developed an integrated framework for multi-object tracking and action recognition in volleyball by building upon YOLOv7 and incorporating a Transformer architecture with a spatiotemporal memory mechanism. The proposed method achieved an average precision of 94.5% on the SportsMOT dataset at an intersection-over-union threshold of 0.5 while maintaining a real-time processing speed of 29.6 frames per second (FPS). Cross-frame trajectory association was further accomplished using a Memory Encoder–Decoder, effectively mitigating identity drift caused by occlusion and similar player appearances. Experimental results demonstrated that the framework achieved Multiple Object Tracking Accuracy, Identification F1 Score, and Higher Order Tracking Accuracy of 94.8%, 72.6%, and 73.7%, respectively, together with a mean Average Precision (mAP) of 90.7% for group activity recognition. However, the performance of this approach still relies heavily on high-quality detection and stable cross-frame association. Its robustness remains to be further validated under real-world conditions involving severe occlusion, camera switching, or significant viewpoint variations.

Compared with other frameworks, some studies have improved detection models at the architectural level to enhance fundamental perception capabilities. For example, Huang et al. (39) proposed a Graph-Embedded YOLO framework by integrating a graph convolutional network into YOLOv4 and combining it with DeepSORT for volleyball trajectory estimation, effectively improving small-object detection and trajectory analysis. This study indicates that incorporating graph-structured priors into the detection stage can improve object representation and trajectory stability in volleyball scenarios, particularly for the ball, which is small and fast-moving. However, such methods mainly focus on object localization and trajectory extraction, and remain limited in capturing action semantics and complex behavioral patterns. Detection and tracking networks provide an important starting point for structuring volleyball match data, but their focus is primarily on perceiving targets rather than understanding behaviors. This limitation has driven subsequent research toward convolutional neural network models that place greater emphasis on visual feature extraction and action semantic representation.

#### 2.2.2 Convolutional neural networks

**Figure 2:**
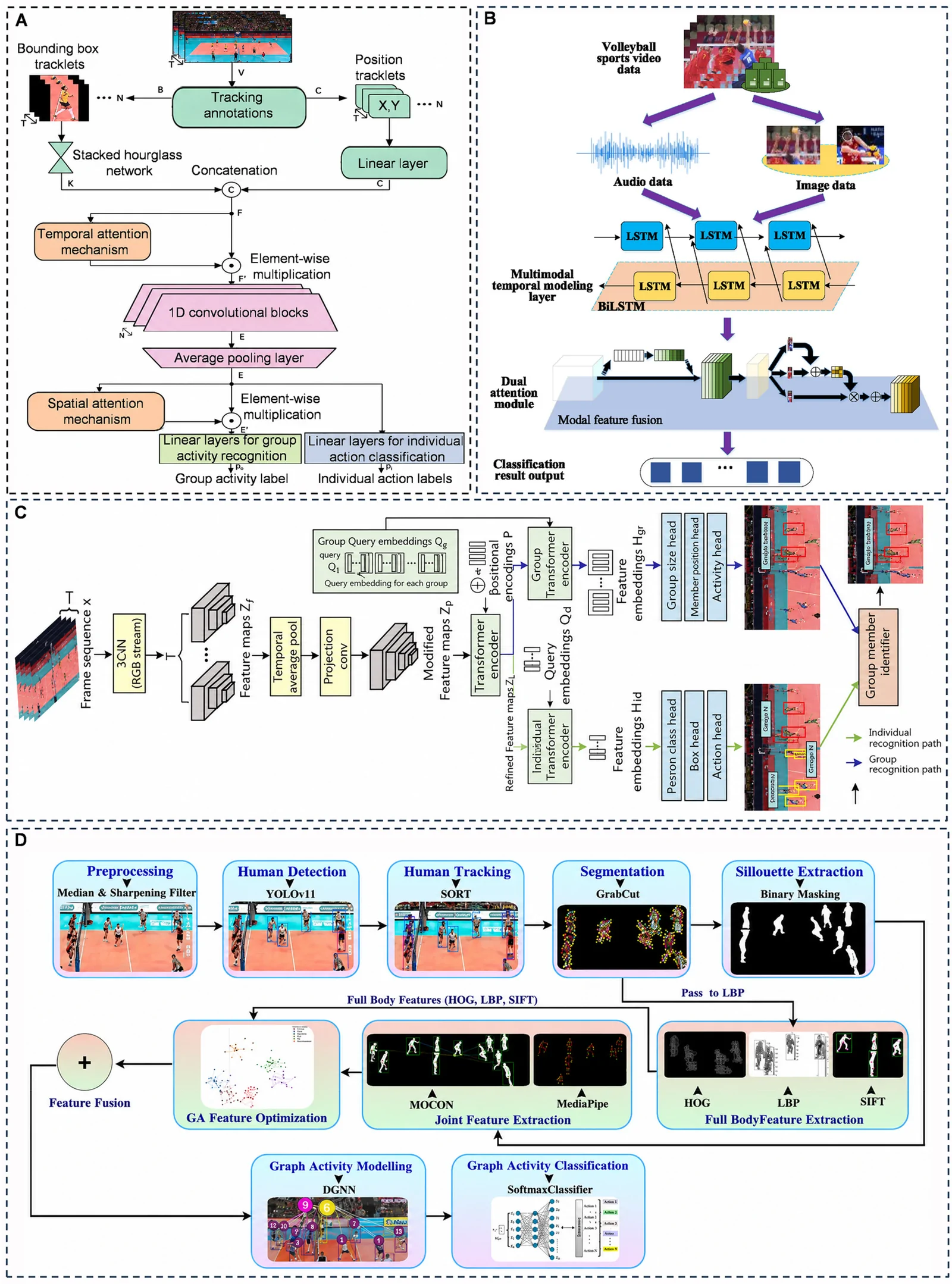
Deep learning-based approaches in volleyball analysis. **(A)** CNN-based group activity recognition model (POGARS) (40). **(B)** Multimodal volleyball video classification based on BiLSTM and attention mechanisms (97). **(C)** Transformer-based group and individual behavior recognition in volleyball (44).**(D)** Group activity recognition using multimodal feature fusion and dynamic graph neural networks (105).

Beyond basic object detection, convolutional neural networks (CNNs) (90–92) and their extensions have been widely applied to visual feature extraction and action recognition in volleyball. Two-dimensional CNNs are primarily used for single-frame or keyframe feature extraction, whereas three-dimensional CNNs enable joint spatiotemporal modeling of video clips, thereby improving the recognition of continuous actions (86). Compared with detection and tracking networks, CNNs place greater emphasis on the semantic representation of actions, making them a critical transition from target perception to action understanding in volleyball analysis. For example, Thilakarathne et al. (40) proposed the Pose Only Group Activity Recognition System (POGARS) (as shown in Figure 2A), by integrating skeletal key points and positional trajectory information with a spatiotemporal attention mechanism. In this framework, CNNs are employed to model the spatiotemporal dynamics of action sequences in volleyball, enabling effective recognition of multi-player cooperative behaviors. Notably, the method is capable of accurately capturing action evolution and group interaction patterns without relying on RGB pixel information, achieving a recognition accuracy of 93.2% on a volleyball dataset.

However, this approach is highly sensitive to the quality of pose estimation; errors in key point detection under conditions such as occlusion, rapid body rotation, or multi-player overlap can significantly degrade subsequent recognition performance. Moreover, while pose and trajectory features effectively capture motion patterns, they remain insufficient for fully representing tactical information and ball possession dynamics. Meanwhile, Wang et al. (41) constructed a volleyball action dataset and employed an improved 3D CNN model for action recognition, increasing accuracy to 88.5% while reducing computational complexity by approximately 33.6%, thereby advancing the transition from manual evaluation to automated recognition. Nevertheless, although 3D CNNs perform well in capturing local spatiotemporal patterns, their modeling windows are typically limited, making it difficult to cover long-term action evolution. Overall, CNNs demonstrate clear advantages in spatial feature extraction and local spatiotemporal modeling, forming a key foundation for volleyball action recognition. However, their ability to model long-term temporal dependencies remains limited, and they are less effective in capturing dynamic interactions among multiple agents.

#### 2.2.3 Recurrent neural networks

To address the limitations of CNNs in temporal modeling, recurrent neural networks (RNNs) (93–95) and their variants, such as long short-term memory (LSTM) networks (96), explicitly model temporal dependencies and play an important role in volleyball action sequence analysis. Compared with CNNs, which focus on local spatiotemporal patterns, RNN/LSTM architectures emphasize sequential context continuity, making them more suitable for action evolution modeling, video classification, and continuous state assessment. For example, Ruiye et al. (97) proposed a multimodal temporal modeling framework based on bidirectional long short-term memory (BiLSTM) with an attention mechanism (Figure 2B). The method extracts visual and audio features and applies bidirectional temporal modeling to capture forward and backward dependencies in action evolution, while a dual-attention module integrating spatial and channel attention enables weighted fusion of cross-modal features. Final classification is obtained via fully connected layers and a Softmax function. The proposed model achieved Top-1 and Top-5 classification accuracies exceeding 95% on the volleyball video classification task, demonstrating the effectiveness of bidirectional temporal modeling and multimodal feature fusion for efficient and accurate clip-level action classification. However, its temporal modeling remains focused on clip-level classification and is still insufficient for capturing long-term dynamics such as tactical organization, rally progression, and multi-agent interactions.

Building upon this, RNN-based approaches have been further extended to tasks such as performance evaluation and action recognition. For example, Dong et al. (98) developed an RNN-based model for evaluating volleyball training performance. The model achieved an *R*^2^ of 0.927 in predicting volleyball training performance, outperforming the conventional LSTM model. This suggests that recurrent architectures can be applied not only to action recognition but also to continuous training state assessment. However, the study focuses primarily on predictive performance, with limited interpretability regarding how internal variables relate to specific training mechanisms and how different indicators influence the output. In addition, Xie et al. (42) combined multi-scale spatiotemporal attention with RNN-based modeling for action recognition, achieving an accuracy of 99.6% on a volleyball dataset. While this result is notable, such high accuracy warrants careful consideration of dataset scale, task complexity, and generalization limits, particularly in cross-scenario, cross-team, and real-match conditions. Furthermore, hybrid models integrating temporal and spatial relational modeling have also been explored. For example, Wang et al. (43) proposed a hybrid framework integrating LSTM and graph convolutional network (GCN) architectures for volleyball pose recognition. The proposed framework achieved a recognition accuracy of 93.68%., demonstrating that coupling temporal and relational modeling enhances representation of complex actions. However, such hybrid models typically introduce increased structural complexity and training cost, posing challenges for practical deployment.

#### 2.2.4 Transformer

To further enhance the modeling of long-range temporal dependencies and complex interaction behaviors, Transformer models (99–101) have been introduced into volleyball behavior analysis. Based on self-attention mechanisms, these models directly capture global dependencies, effectively overcoming the limitations of RNNs in long-sequence modeling. For multi-agent team sports such as volleyball, the advantages of Transformers extend beyond temporal integration to the unified modeling of individual actions, group behaviors, and on-court relationships within a single framework. For example, Chen et al. (44) proposed a Transformer-based framework that integrates individual and group modeling for multi-player behavior recognition and tactical analysis in volleyball (Figure 2C). Video sequences are used as input, with spatiotemporal features extracted via 3D convolution, followed by pooling and projection to obtain compact representations. A Transformer module is then introduced, combining group-level and individual-level queries for global modeling. The method achieves a group activity recognition mAP of 92.8% and an F1-score of up to 92.1% on the Volleyball dataset, significantly improving performance in complex multi-agent interaction scenarios. However, such Transformer-based frameworks typically require large-scale, high-quality annotated datasets, which remain limited in the volleyball domain. Moreover, although the global attention mechanism facilitates the integration of long-range dependencies, it may also introduce redundant associations, and its ability to selectively capture truly critical interactions still requires further improvement.

Building upon this, subsequent studies have sought to optimize Transformer architectures from the perspective of attention modeling. For instance, Kim et al. (45) proposed the GL-Transformer++ model, which jointly models spatial and temporal attention to efficiently capture multi-player interactions, achieving superior performance in group activity recognition tasks. By adapting the self-attention mechanism to domain-specific characteristics of sports scenarios, this approach improves task suitability. However, such enhancements often introduce more complex module stacking and parameterization, and the trade-off between model interpretability and deployment efficiency remains unresolved. Transformers have clear advantages in modeling long-range dependencies and integrating global interactions, promoting volleyball analysis toward higher-level tactical behavior understanding. However, issues such as structural complexity, high data requirements, and insufficient explicit representation of relationships have not yet been fully resolved, leading to a further shift toward graph-based modeling approaches.

#### 2.2.5 Graph neural networks

To address the limitations of Transformers in explicitly modeling multi-agent relationships, graph neural networks (GNNs) (102–104) construct graph structures in which players are represented as nodes and their interactions as edges, enabling explicit modeling of multi-agent relationships and providing a more structured solution for volleyball tactical behavior analysis. Compared with attention-based methods that implicitly learn interaction patterns, GNNs emphasize the explicit representation of relational structures among players, making them particularly suitable for team sports characterized by strong collective coordination, such as volleyball. For example, Zahra et al. (105) proposed a volleyball group activity recognition framework that integrates multimodal features with a dynamic GNN (Figure 2D). The method first performs player detection and tracking using YOLOv11 and SORT, and then extracts appearance, skeletal, and motion features using HOG, LBP, SIFT, MediaPipe, and MOCON. Experimental results show that the model achieves 94.5% accuracy, an mAP of 94.2%, and a mean per-class accuracy of 93.8% on a volleyball dataset, with an inference time of 0.18 s per frame, demonstrating strong group behavior recognition capability. This framework integrates visual features, skeletal information, dynamic graph structures, and temporal modules, effectively capturing the multi-dimensional characteristics of complex group behaviors in volleyball. However, while such multi-module architectures achieve strong performance, they also significantly increase system complexity and computational cost.

Subsequent research has further optimized GNNs from the perspectives of graph representation and modeling. Liu et al. (46) proposed a visual–semantic graph neural network, achieving approximately 93% accuracy on the Volleyball dataset, indicating that incorporating semantic information enhances graph representation capability; however, its performance still depends on the quality of front-end visual features and graph construction strategies, and inaccurate node features or edge definitions may limit the effectiveness of graph inference. In addition, Jiang et al. (47) introduced a local–global context graph reasoning model, achieving 92.7% accuracy, demonstrating that jointly modeling local interactions and global context improves group activity recognition; nevertheless, such approaches rely heavily on context design, and their transferability and cross-scenario robustness require further validation. Furthermore, Wu et al. (106) proposed a multi-view part-based graph representation for fine-grained modeling, improving recognition accuracy to 94.8%. This result suggests that fine-grained, part-level graph representations can enhance the recognition of complex action relationships; however, increased granularity also incurs higher costs in feature extraction and graph construction, highlighting the trade-off between model complexity and practical efficiency. Overall, GNNs explicitly model multi-agent interactions, effectively compensating for the limitations of Transformers in structural representation and demonstrating strong relational modeling capability in complex sports scenarios. However, existing GNN approaches generally involve long model pipelines, strong dependence on front-end detection and feature extraction, limited cross-scenario generalization, and high deployment cost. Achieving more efficient, robust, and interpretable models while preserving relational reasoning capability remains a key challenge for future research.

## 3 Wearable sensing-based volleyball analysis

**Figure 3:**
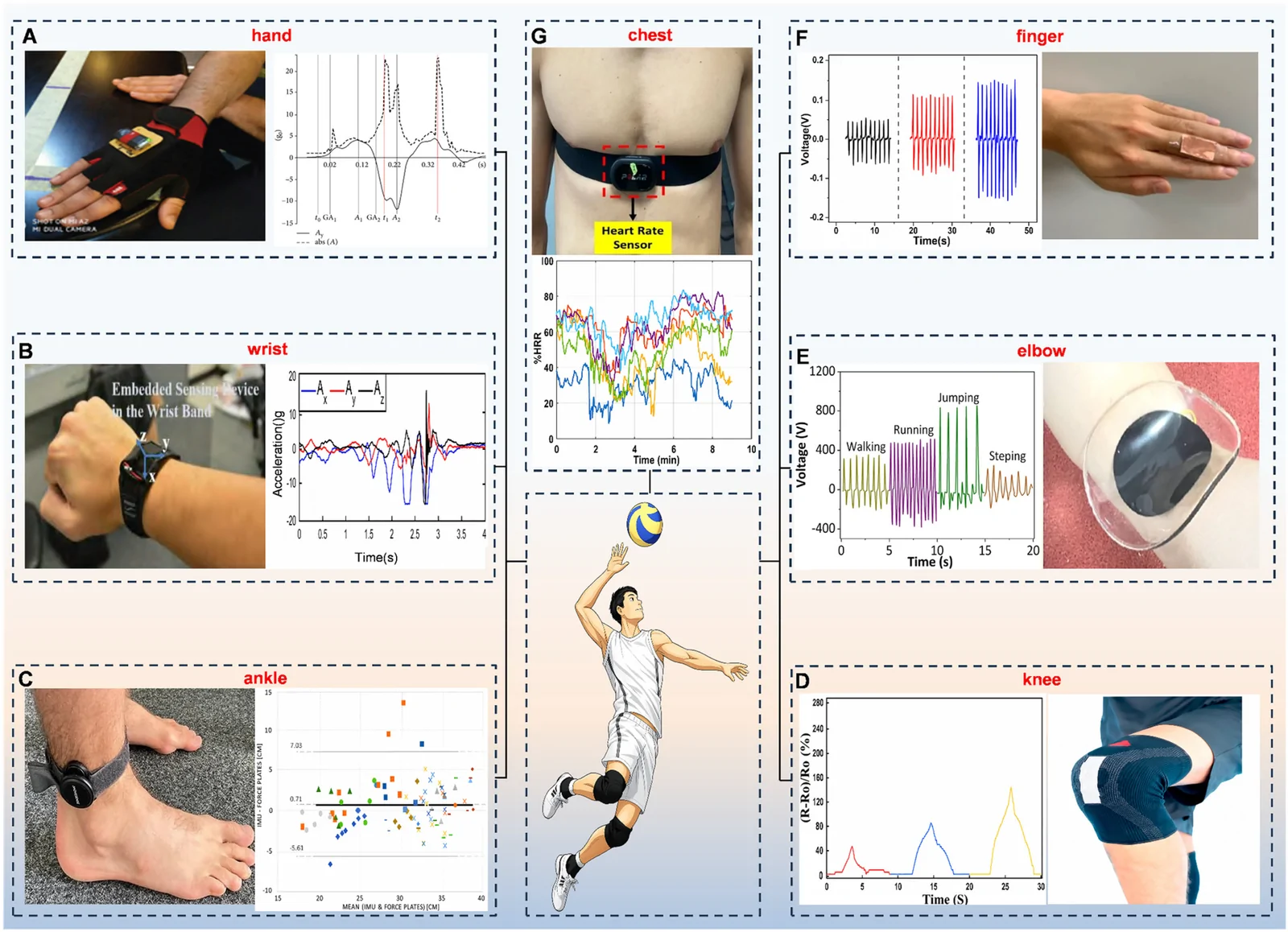
Wearable sensor-based applications in volleyball. **(A)** IMU-based rapid hand movement testing system and the corresponding acceleration (*Ay*) and absolute acceleration (Abs) signals (110). **(B)** Wrist-mounted IMU and the corresponding triaxial acceleration signals (*Ax*, *Ay*, and *Az*) acquired during volleyball spiking (111). **(C)** IMU sensor mounted at the lateral malleolus and Bland–Altman agreement analysis between the measured jump height and the force plate (gold standard) (115). **(D)** Knee-mounted flexible strain sensor and the corresponding relative resistance ((*R* − *R*_0_)/*R*_0_) response curves at different knee flexion angles (116). **(E)** Elbow-mounted flexible self-powered triboelectric sensor and the corresponding output voltage signals during different movement conditions, including walking, running, jumping, and stepping (117). **(F)** Finger-mounted flexible self-powered sensor and the corresponding output voltage signals under different finger bending conditions (118). **(G)** Chest-worn heart rate sensor and the corresponding %HRR profile calculated from the acquired physiological data (120).

In volleyball, characterized by high intensity and intermittent dynamics, athletes frequently perform jumps, rapid directional changes, and explosive technical actions. As a result, both physical workload and execution quality play critical roles in determining match outcomes and injury risk (107). Accordingly, the accurate quantification of workload, technical performance, and physiological state has become a key issue in scientific training and match analysis. In recent years, the development of wearable sensing technologies has enabled real-time data acquisition and objective analysis in volleyball, facilitating a shift from experience-driven to data-driven performance evaluation. Among these technologies, IMUs, owing to their sensitivity to rapid dynamic movements and flexible deployment, have become one of the primary tools for motion data acquisition and analysis in volleyball (51, 108, 109).In volleyball, the upper limbs and hands serve as the primary execution units for technical actions, and their movement characteristics directly influence key skills such as serving, spiking, and blocking, making them a major focus of sensor-based monitoring. For example, Marković et al. (110) developed a glove-based IMU system (Figure 3A) to capture real-time hand acceleration, angular velocity, and orientation data. By extracting temporal and kinematic features (e.g., *t*_1_, *t*_2_, *A*_1_, and *A*_2_), the system was able to distinguish athletes of different skill levels, achieving a classification accuracy of approximately 65.7%, which increased to over 80% among youth athletes. However, the effectiveness of such systems largely depends on standardized testing tasks and controlled experimental conditions, and their generalization capability in real training and competition environments remains to be further validated.

Compared with glove-based systems, wrist-mounted IMU devices offer better wearability in practical scenarios. Wang et al. (111) developed a microelectromechanical system (MEMS)-based wrist IMU (Figure 3B) capable of continuously collecting motion data during spiking, and combined principal component analysis (PCA) with SVM to classify athlete levels, achieving an accuracy of approximately 94%. The system effectively captures key kinematic features and establishes a complete mapping from motion signals to skill evaluation, demonstrating the potential of single-point inertial sensing in low-burden scenarios. However, although wrist-based deployment improves usability, it cannot fully represent the coordination among the trunk, shoulder–elbow–wrist chain, and lower limbs, leading to clear information gaps in complex action analysis and overall workload characterization Accordingly, research has gradually shifted from single-task recognition to quantitative modeling of movement load. Damji et al. (112) further incorporated landing impact, quantified by peak resultant acceleration, into jump load assessment. Compared with conventional metrics based solely on jump count and jump height, this approach provided a more comprehensive characterization of landing load. However, it still relied exclusively on data acquired from a single IMU mounted at the lumbar region near the L3/L4 vertebral level and therefore remained insufficient to capture higher-level information, such as movement quality, internal fatigue, and physiological responses. Mackay et al. (113) further validated the reliability of the Catapult Vector S7 wearable inertial measurement system for overall movement load assessment. The system integrates a tri-axial accelerometer, gyroscope, and magnetometer, and the results demonstrated that it can reliably monitor movement load during field-based sports activities as a wearable microtechnology device. However, its performance in fine-grained action recognition remained limited, suggesting that device availability does not necessarily translate into behavioral interpretability. Lima et al. (114) continuously monitored jump frequency and jump height using a wearable IMU positioned at the hip and identified a significant correlation between these two variables, thereby contributing to the establishment of the relationship between training load and performance. Although this work helps link inertial sensing with training outcomes, the analysis remains largely at the level of correlation, and provides limited insight into the dynamic variations of load–performance relationships across different player positions, training stages, and fatigue conditions.

Furthermore, to meet the demands for improved accuracy and real-time performance in load quantification, IMU systems have gradually evolved toward multi-point collaborative sensing. Schleitzer et al. (115) developed a jump recognition system based on ankle and trunk IMU signals (Figure 3C), enabling automatic detection and height estimation of jumps in beach volleyball. The system achieved recognition accuracies of 100% in laboratory settings and over 95% in real-world scenarios, with high consistency compared to force plate measurements (intraclass correlation coefficient ≈ 0.94), demonstrating its reliability in workload quantification. However, with increasing monitoring duration and exercise intensity, the dependence of conventional IMU systems on external power supply has become a limiting factor. Building on flexible sensing technologies, self-powered mechanisms have further expanded the applicability of such systems in complex motion scenarios. For example, Raza et al. (116) developed a flexible textile sensor based on an laser-induced graphene (LIG)/polydimethylsiloxane (PDMS) structure, designed for key regions such as the knee joint that undergo large deformation and repeated bending (Figure 3D). The sensor enables simultaneous strain and pressure sensing, exhibiting good linearity (*R*^2^ ≈ 99.1%), high sensitivity (gauge factor ≈ 3.54), stable response within a range of 0–220 kPa, and sustained performance after more than 10,000 cycles. In addition, Yang et al. (117) developed a PDMS/MXene-based triboelectric nanogenerator (TENG) device that simultaneously harvests human biomechanical energy and functions as a self-powered motion sensor (Figure 3E). Benefiting from the enhanced charge trapping and transport capabilities of MXene, the device achieved a maximum output power of approximately 11.27 mW, representing an approximately 11-fold improvement over conventional PDMS-based systems and enabling the integrated realization of energy harvesting and motion sensing.

Building upon this, to achieve more refined characterization of volleyball hitting actions, self-powered flexible sensing has been extended to key regions such as the fingers. Liu et al. (118) developed a flexible self-powered sensor based on piezoelectric poly(vinylidene fluoride) (PVDF) that can be attached to the fingers and elbows for continuous acquisition of joint bending signals without the need for an external power supply while supporting wireless data transmission (Figure 3F). Although self-powered technologies improve system endurance, comprehensive characterization of complex motion still relies on multimodal sensing. Marotta et al. (119) integrated data from an IMU worn at the lumbar region with measurements acquired from a plantar pressure platform to enable a comprehensive assessment of changes in athletic performance. Their findings demonstrated that multimodal data integration provides a more comprehensive characterization of movement function. In addition, the trunk region serves as a key site for internal load monitoring. For example, Aydın et al. (120) developed a multisensor chest-worn system that integrates multimodal physiological and movement data for comprehensive training load assessment (Figure 3G). The system simultaneously quantified internal load using percentage heart rate reserve (%HRR) and training impulse (TRIMP), while external load was evaluated using PlayerLoad (PL) and accumulated PlayerLoad (APL), enabling a comprehensive assessment of training load across multiple athletes during team-based training sessions.

## 4 Volleyball analysis and decision-making applications

As volleyball continues to evolve toward a data-driven and intelligent paradigm, the application scope of AI has expanded beyond conventional post-match analysis to encompass higher-level tasks, including training regulation, match understanding, and decision support. Given the high intensity, rapid pace, and pronounced multi-agent interactions inherent to volleyball, traditional experience-driven approaches are insufficient to continuously and comprehensively characterize the evolution of player behavior, workload dynamics, and tactical organization. Consequently, leveraging multimodal data for accurate perception, objective performance evaluation, and evidence-based decision-making has become a major research focus in modern volleyball analytics. Depending on the analytical objective and application scenario, current research can be broadly categorized into five representative tasks, including action recognition (e.g., serving, passing, spiking, and blocking), event detection (e.g., point scoring, errors, and offense–defense transitions), performance evaluation (e.g., quantification of technical proficiency, physical condition, and training load), training decision-making and tactical optimization (e.g., training planning, lineup adjustment, and tactical strategy development), and match outcome prediction, together with their representative wearable sensing systems, data sources, analytical methods, model performance, and major advantages and limitations (see Table 2).

**Table 2:** Characteristics, advantages, and limitations of representative wearable sensing technologies for volleyball performance monitoring.

Volleyball performance parameter	Data source/Sensor	Analysis method	Model performance	Advantages	Limitations	Refs.
Ball trajectory tracking and technical action recognition (serving, receiving, setting, and spiking)	Event camera	Event vision-based ball trajectory tracking and action classification	Classification accuracy: serving 99%, spiking 97%, setting 82%, receiving 67%; satisfies real-time processing requirements (1.144 s per video)	Fully exploits the high temporal resolution and motion perception capability of event cameras, enabling accurate ball tracking and action recognition for video playback and performance analysis	Primarily relies on ball trajectory for action classification; receiving and setting are susceptible to occlusion; limited generalization to complex multi-player interaction scenarios	(121)
Jump performance assessment (serving, spiking, and blocking)	Wearable IMU	IMU-based automatic jump detection algorithm	Jump detection sensitivity: referees 96.29%, male athletes 97.20%, female athletes 94.56%; blocking 97.25%, other jumps 99.10%, spiking 95.75%, serving 94.69%	Automatically quantifies jump workload with high detection accuracy, enabling real-time match monitoring while reducing manual video analysis	Detection accuracy is influenced by individual differences, sex, and technical actions; algorithm optimization is required for different athlete populations	(122)
Rapid upper-limb functional assessment (spiking and serving arm swing)	Wearable IMU	IMU-based motion feature extraction and analysis	Overall classification accuracy: 65.7%; youth players 80.6%; national team players 70.6%	Effectively evaluates rapid upper-limb performance across different skill levels, supporting training monitoring and talent identification	Practical applicability in real-match scenarios requires further validation	(110)
Technical action recognition and training feedback (setting, passing, and spiking)	IMU, pressure-sensitive flooring, and video data	Multimodal data fusion with CNN-based action recognition	CNN: 10-fold cross-validation accuracy 92.78%, UAR 78.71%, mean F1-score 81.72%; ADR: accuracy 96.24%, UAR 74.47%	Integrates multimodal sensing information for accurate technical action recognition, enabling automatic labeling and real-time training feedback to support performance analysis and interactive coaching	Action recognition is affected by inter-individual variability; LOSO validation performance declines; continuous recognition accuracy for the complete Bump–Set–Spike sequence remains limited	(6)
Jump detection and jump height assessment (blocking, spiking, and serving)	Chest- and ankle-mounted IMUs	MATLAB-based IMU jump detection algorithm	Jump recognition accuracy: 95.9%; laboratory: 100%; jump height reliability: test ICC = 0.946, retest ICC = 0.937	Enables automatic monitoring of volleyball-specific jump frequency and height, making it suitable for training load monitoring and athletic performance evaluation	Relies on preset threshold-based algorithms and requires further optimization for athletes with different technical movement patterns	(115)
Spiking technique evaluation (technical proficiency)	Wrist-mounted IMU	SVM	Mean classification accuracy: 94%	Enables rapid assessment of technical proficiency across different skill levels using a single wearable sensor, facilitating practical deployment	Limited to spiking analysis; evaluated with a small sample size (10 athletes); real-time feedback capability requires further validation	(111)
Technical action recognition (serving and blocking)	Five Xsens MTw Awinda IMUs	KNN	KNN (k = 5): four-class accuracy 66.08%; ten-class accuracy 92.39%	Integrates multi-IMU sensing for synchronized motion recognition, enabling optimization of technical movement quality and supporting training analysis	Evaluated only for serving and blocking; relies on wearable sensors; generalization to complex scenarios and other technical actions requires further validation	(123)
External training load assessment before matches (total distance, jump count, acceleration, passing actions, etc.)	MEMS-based local positioning system	LPS-based training load monitoring and quantitative analysis	Continuous monitoring across 18 official matches revealed significant differences in total external load between starters and non-starters (*P* < 0.001)	Enables quantitative assessment of external training load before matches, identifying key load characteristics to support individualized training programs	Monitors only external load without integrating internal physiological load; evaluated exclusively in a women's collegiate volleyball team, requiring validation across broader athlete populations	(124)
Team training monitoring and internal/external load assessment (HR, HRV, body temperature, Player Load, TRIMP, etc.)	Polar H10 chest strap, CORE body temperature sensor, and inertial sensor	Multisensor fusion for real-time monitoring and load quantification	Real-time monitoring over six consecutive official matches enabled calculation of HR, TRIMP, Player Load, and Accumulated Player Load	Enables real-time monitoring of internal and external training loads, integrating physiological and biomechanical indicators to provide immediate feedback for training load management	Evaluated in only six official matches with a relatively small sample size; further validation in larger cohorts is required	(120)
Match tactical decision-making and offensive strategy optimization	FIVB match statistics, attack quality, attack transition, and rally-state information	CNN combined with reinforcement learning	Match outcome prediction accuracy: 95.4%; win-rate prediction accuracy: 89.3%; attack efficiency improved from 2.8 to 3.0 (387 vs. 1,456 epochs); real-time decision latency: 23.7 ms	Enables rapid tactical decision-making in real time, improving offensive efficiency and tactical adaptability while supporting intelligent coaching decisions	Relies on high-quality match data; limited adaptability to complex tactical scenarios; requires further validation in real competitive environments	(125)
Match technical statistics analysis and tactical decision optimization	DataVolley match technical statistics	Machine learning and gray relational analysis	Strong consistency with expert evaluations (ARI = 0.66–0.82); AB correlation coefficient = 0.86; FBSD = 0.65; AST = 0.70; significant correlation with wins and losses	Integrates match visualization with clustering analysis and tactical evaluation, supporting tactical interpretation and decision optimization	Relies on manually collected technical statistics; affected by subjective indicator selection; integration with automated video analysis remains limited	(8)
Match outcome prediction and key determinant analysis (spiking, blocking, serving, and defensive performance indicators)	DataVolley match statistics and FIVB official match database	GRA combined with machine learning	MSE = 0.002; MAE = 0.0322; *R*^2^ = 0.8497; MAPE = 4.77%; prediction error reduced by 5.30%; *R*^2^ = 0.743	Accurately identifies key determinants of match outcomes, enabling match result prediction and providing quantitative support for coaching decisions and tactical design	Relies on manually annotated match statistics; limited sample size; model generalization requires validation using larger datasets	(34)
Spiking performance assessment	Standard RGB video sequences	CNN	Classification accuracy: 99.16%; Top-2 accuracy: 100%; MAE = 0.0003°; inference speed: 24.8 ms/frame (30 FPS)	Enables accurate estimation of spiking performance from monocular video, providing real-time quantitative feedback without wearable sensors	Primarily trained on elite athlete data; requires further validation across different player populations and tactical scenarios	(126)

ADR, automatic data recording; ARI, adjusted Rand index; AST, average silhouette test; FBSD, feature-based similarity distance; FPS, frames per second; GRA, gray relational analysis; ICC, intraclass correlation coefficient; LOSO, leave-one-subject-out; MAE, mean absolute error; MAPE, mean absolute percentage error; MSE, mean squared error; UAR, unweighted average recall.

### 4.1 Match and training analysis in volleyball

During volleyball matches and training, athletes frequently perform high-intensity actions such as spiking, blocking, jumping, and rapid movement, resulting in motion patterns characterized by fast tempo, complex interactions, and strong temporal dependencies. Therefore, the continuous, precise, and automated recognition of key actions, match events, and behavioral changes during training has become a fundamental component of data-driven analysis and intelligent decision-making in volleyball. Current research has gradually established a technical pipeline progressing from wearable sensing to action and event recognition, and further to training analysis, with ongoing developments in computer vision, wearable sensing, and multimodal data fusion. For example, in early work, Yamane et al. (121) proposed a real-time ball tracking and action recognition method based on an event camera. By leveraging the camera's high temporal resolution and low motion blur characteristics, the proposed method enabled continuous tracking of high-speed volleyball trajectories together with the recognition of key technical actions, including serving, serve reception, setting, and spiking. While event-based vision demonstrates clear advantages in high-speed target perception, it primarily focuses on object-level detection and tracking, and remains limited in handling complex occlusion, multi-agent interactions, and stable capture of fine-grained individual actions in training scenarios. The introduction of wearable sensing technologies helps compensate for some of these limitations in individual motion perception. For instance, Marzano-Felisatti et al. (122) deployed the WIMU PRO™ wearable inertial measurement system across 42 official matches to automatically detect and classify athletes’ jumping events. The results demonstrated a jump detection sensitivity of 96.29%, while the system accurately distinguished different types of volleyball-specific jumps, including serves, spikes, and blocks, thereby providing a reliable basis for jump load monitoring and training decision-making under competitive match conditions. However, this method mainly focuses on a limited set of high-frequency actions, such as jumping, and remains less effective in monitoring spiking, blocking, and complex sequential actions.

**Figure 4:**
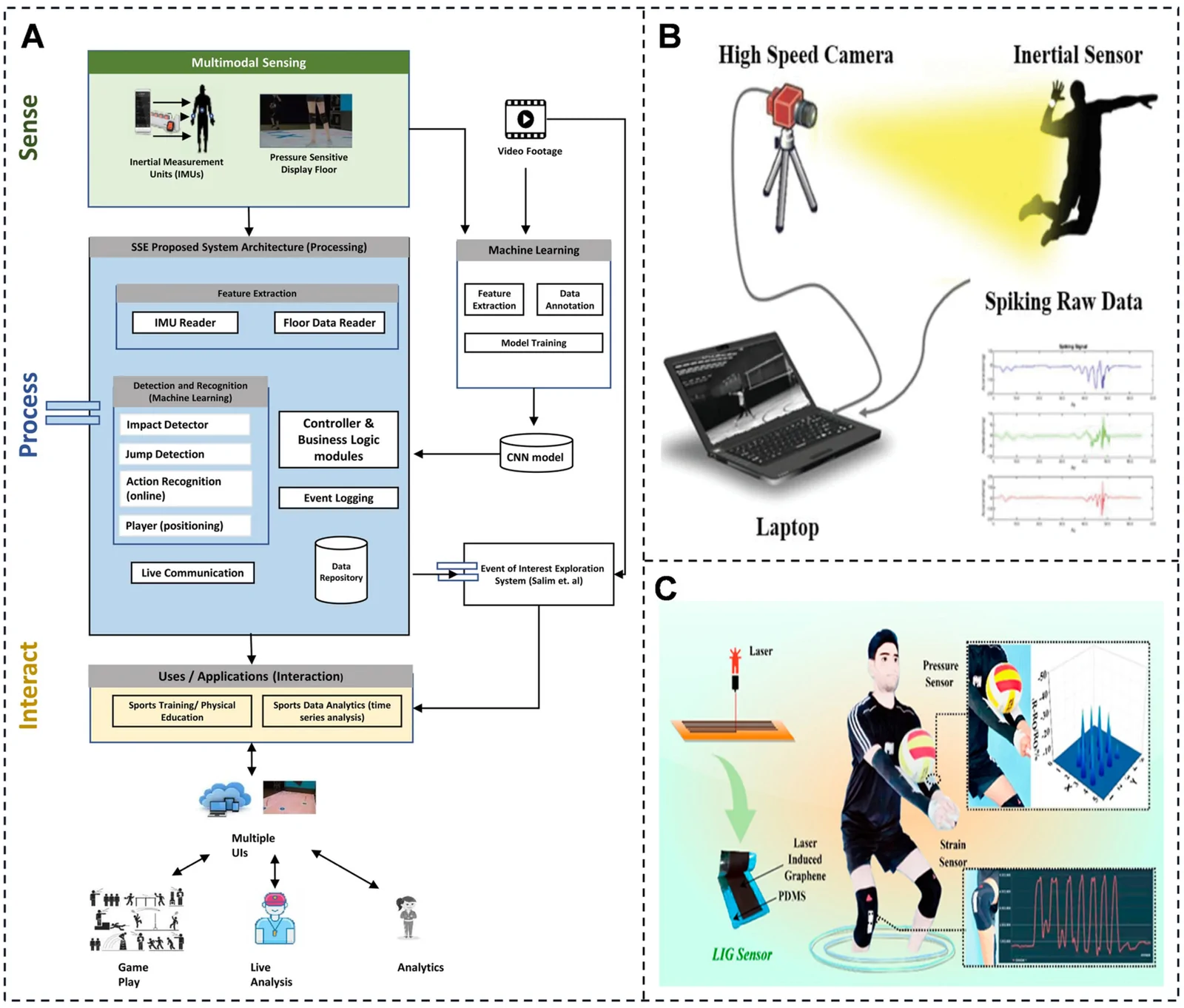
Wearable sensor-based framework for volleyball match and training analysis. **(A)** Modular architecture of the SSE volleyball training system (6). **(B)** Data acquisition system for spiking motions based on inertial sensors and a high-speed camera (111). **(C)** Application of a flexible multifunctional wearable sensing system based on LIG in volleyball (116).

Building upon this, multimodal data fusion has gradually become an important direction for improving match and training analysis. Salim et al. (6) developed a multimodal intelligent analysis system for volleyball training that integrates data from IMUs, a pressure-sensitive floor, and video recordings to enable synchronized acquisition of multi-site movement information at the sensing layer (Figure 4A). Machine learning algorithms were subsequently employed to perform action recognition and structured recording of key events, thereby supporting training analysis and real-time feedback. The results demonstrated that the proposed system enabled automatic recognition of volleyball-specific actions, achieving an unweighted average recall (UAR) of 78.71% under 10-fold cross-validation, thereby providing effective technical support for action analysis and interactive feedback during volleyball training. While such systems enhance information richness, they also increase the complexity of device deployment, time synchronization, and data fusion, and their cost and scalability in real training and competition scenarios still require further balance. Notably, Wang et al. (111) proposed a lightweight action sensing method based on a single wrist-mounted IMU (Figure 4B). By collecting tri-axial acceleration and angular velocity signals from the wrist, extracting time- and frequency-domain features, and synchronizing with high-speed camera recordings for annotation, the method achieves high-accuracy recognition of spiking actions. While maintaining strong performance, it significantly simplifies the system structure, reflecting a trend toward lightweight and wearable solutions in volleyball action recognition.

To achieve multimodal motion sensing and enable a more comprehensive characterization of human movement, flexible electronic sensing technologies have been increasingly adopted in volleyball applications. For example, Raza et al. (116) proposed a wearable flexible sensor based on laser-induced graphene (Figure 4C), integrating strain and pressure sensing units into flexible textiles to enable simultaneous acquisition of joint motion and contact impact, thereby supporting fine-grained recognition of actions such as spiking, receiving, and blocking. However, the stability, durability, and deployment efficiency of such sensors under conditions of heavy sweating, repeated friction, and prolonged wear remain insufficiently validated. With the development of deep learning, unified modeling of multiple targets, actions, and group behaviors has become feasible. For instance, Shih et al. (127) developed a volleyball action detection model based on the Detection Transformer architecture, which enables joint recognition of individual actions and group behaviors in match videos. The model demonstrates high detection accuracy and real-time performance in complex scenarios, providing an effective approach for unified modeling of multi-agent interactions. This work further promotes the transition of volleyball analysis from isolated action recognition toward group behavior understanding.

### 4.2 Athlete performance quantification and workload assessment

**Figure 5:**
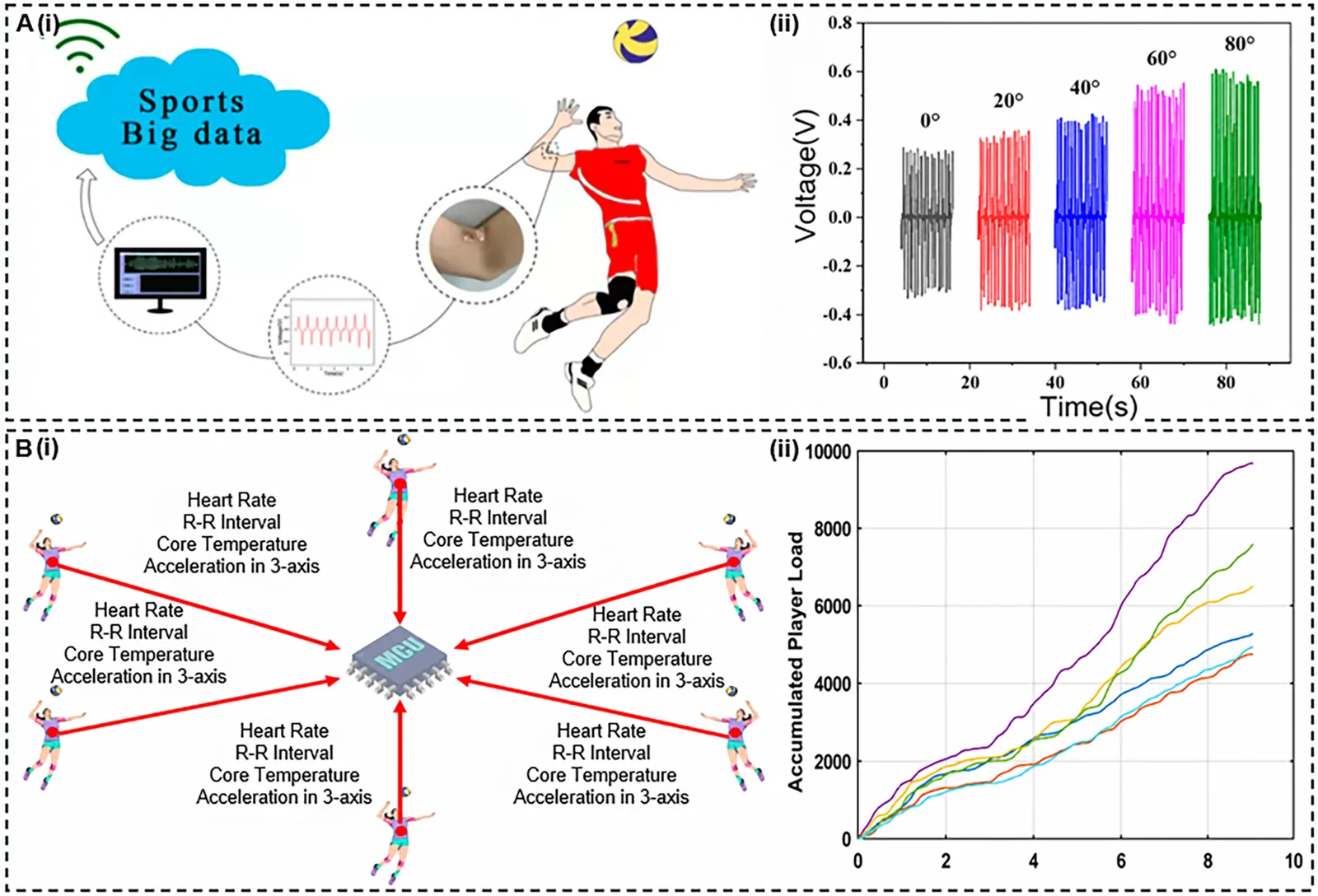
Wearable sensor-based quantification of athlete performance and training load assessment in volleyball. **(A)** Self-powered wearable sensing system based on piezoelectric PVDF film for motion monitoring and technique evaluation. (i) Application of the wearable sensor in volleyball; (ii) Signal responses under different bending angles (118). **(B)** Multisensor fusion system for volleyball performance monitoring. (i) Synchronous acquisition of physiological and motion parameters, including heart rate, *R*–*R* interval, core body temperature, and tri-axial acceleration; (ii) APL during training (120).

Athlete performance evaluation is a central issue in sports science research and practice. Due to the high-frequency jumping, short-duration explosive actions, and clear positional specialization in volleyball, a single metric is insufficient to comprehensively reflect an athlete's competitive state. Therefore, monitoring technical performance, workload, and physiological status through multi-source data has become essential for scientific training and precise evaluation. In recent years, AI methods, combined with wearable sensing, kinematic analysis, and data modeling, have provided new pathways for multidimensional performance quantification. For example, Liu et al. (118) developed a self-powered wearable sensing system for volleyball technical performance evaluation (Figure 5A). By monitoring finger and elbow bending during spiking, the system enabled quantitative evaluation of movement amplitude and technical execution, thereby providing an effective approach for the objective assessment of volleyball technical performance. However, such approaches primarily focus on local movements and remain limited in reflecting overall performance, action continuity, and tactical role differences in real match contexts. To overcome the limitations of single-action analysis, Jia et al. (123) deployed IMUs at multiple body locations and applied machine learning algorithms to recognize and analyze various volleyball-specific techniques, including forearm passing and blocking, thereby establishing a mapping between sensor-derived data and athletic performance. The proposed method enabled continuous monitoring of technical actions throughout the movement process, extending performance evaluation from instantaneous action analysis to dynamic process characterization. The proposed method achieved an accuracy of 99.56%, demonstrating the feasibility of wearable inertial sensing for sports performance analysis. Nevertheless, these approaches still rely primarily on inertial signals to characterize observable movement patterns, with limited consideration of underlying physiological fatigue, internal load, and neuromuscular states.

As the focus extends from individual actions to the overall training process, performance evaluation has gradually shifted toward the quantification and regulation of movement load. For example, Altundağ et al. (124) employed a local positioning system integrated with miniature sensors to continuously monitor the movement behaviors of elite female volleyball players during the pre-match warm-up phase. By analyzing positional data, speed, acceleration, jump count, and metabolic load, the study revealed position-specific differences in movement load among players. The results further demonstrated significant variations in movement load characteristics across playing positions, which were closely associated with their respective tactical roles during competition. Similarly, Aydın et al. (120) developed a performance tracking system for volleyball team training by integrating multimodal physiological and movement data (Figure 5B), simultaneously quantifying internal load using %HRR and TRIMP, while evaluating external load using PL and APL, thereby enabling comprehensive assessment of internal and external loads across multiple athletes during team-based training. The results show that different athletes exhibit distinct load accumulation patterns under the same training conditions, reflecting individual differences in response to training stimuli. This multimodal approach improves the granularity of training monitoring. In addition to load quantification, changes in movement quality and functional status are also important components of performance evaluation. For example, Kim et al. (128) combined a three-dimensional motion capture system with force plates to conduct a four-week training intervention analysis of the kinematics, kinetics, and knee joint loading during the take-off and landing phases of volleyball spiking. The results indicate that vertical ground reaction force during landing was significantly reduced, while force output patterns during take-off were optimized, demonstrating that integrating biomechanical metrics with functional state monitoring helps to better understand training effects.

### 4.3 Training and tactical optimization

Compared with *post hoc* evaluation of training outcomes, dynamically adjusting technical execution and tactical decisions based on real-time or near-real-time information during training has become an important direction in intelligent volleyball analysis. In this context, the integration of multi-source data and AI has driven training processes from experience-based approaches toward data-supported and model-assisted decision-making. Traditional training relies heavily on coaches’ subjective observation and correction of technical details, making it difficult to achieve continuous, quantitative, and individualized guidance. To address this, Yang et al. (117) further applied a PDMS/MXene-based self-powered TENG to multi-joint motion monitoring. By deploying sensors on the fingers, wrist, elbow, and knee, the system simultaneously recorded coordinated joint movements during volleyball spiking. The characteristic electrical signals generated by different joints enabled quantitative characterization of force-generation timing and movement rhythm throughout the spiking motion, thereby providing an objective assessment of inter-joint coordination and a basis for personalized technical feedback. However, such systems remain primarily focused on device feasibility and motion monitoring, and are still distant from forming systematic feedback frameworks for coaching decision-making. In tactical decision-making, compared with single-action optimization, the problem involves multi-agent coordination, high-dimensional state spaces, and dynamic interactions, resulting in substantially increased complexity. Cai et al. (125) proposed an AI-based tactical decision-making framework that integrates quantum feature encoding with neural networks to optimize tactical decision-making in multi-agent volleyball scenarios. By improving the computational efficiency of complex decision-making tasks, the framework enables real-time tactical analysis, player positioning analysis, and tactical strategy optimization during matches, thereby providing technical support for coaches in tactical decision-making. However, such methods are typically built upon strong modeling assumptions and idealized environments, and the extent to which the resulting strategies can be directly translated into real-world coaching decisions and player execution remains uncertain.

**Figure 6:**
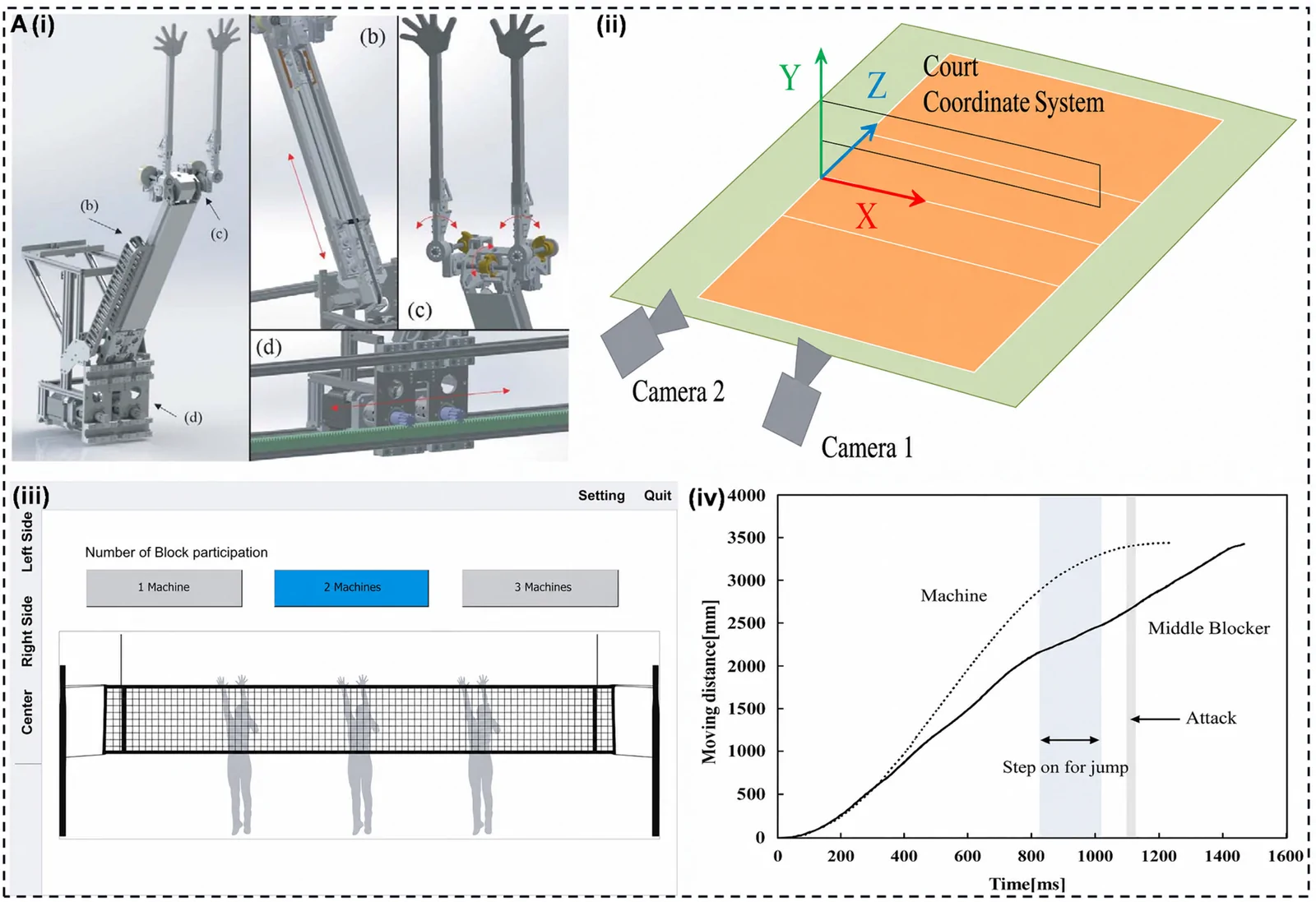
Wearable sensor-based training monitoring and tactical optimization in volleyball. **(A)** Blocking robot system for offensive training and tactical optimization in volleyball. (i) Mechanical structure of a single blocking robot; (ii) 3D trajectory estimation based on dual cameras and court coordinate system; (iii) Coach-side user interface for setting blocking parameters (number of blockers, positions, jump height, arm angle, and timing); (iv) Comparison of athletes’ lateral displacement over time (129).

If the outputs of AI-based tactical models cannot be effectively understood and applied, their practical value will be significantly limited. Accornero et al. (8) developed a volleyball data analysis framework by integrating match technical statistics, rally-level indicators, and visualization into a unified system. In their study, the visualization of match tempo, scoring patterns, and key technical elements allows model outputs to more directly support coaching decisions, enabling analytical results to be effectively incorporated into training adjustments and tactical planning. This indicates that the value of AI in training decision-making depends not only on predictive accuracy, but also on whether the results are presented in a way that aligns with coaching cognition and application needs. In addition, Sato et al. (129) developed a blocking robot system for volleyball offensive training (Figure 6A), which simulates real blocking behaviors to provide athletes with a stable and repeatable training environment. The system includes a multi-degree-of-freedom blocking unit capable of movement, jumping, and arm adjustment, and models real-game behavior by capturing three-dimensional motion trajectories of elite athletes using a dual-camera system. Experimental results show that the system can match or even exceed human players in movement speed and timing control, while continuously providing high-intensity opposition during training.

### 4.4 Match outcome prediction

Compared with interpreting and optimizing existing behaviors, predicting match progression and outcomes based on historical and real-time data, and conducting scenario simulation accordingly, has become an important extension of intelligent volleyball analysis. In this context, research has evolved from early outcome estimation to encompass process modeling, individual analysis, physical characterization of actions, and multi-agent simulation. For example, Ming et al. (34) identified multiple technical indicators, including spiking success rate, blocking height, and defensive performance, as key determinants of volleyball match outcomes. Based on these multidimensional technical indicators, the proposed model enabled quantitative prediction of pre-match winning probability. However, such models typically rely on pre-match statistics and historical data, and thus mainly reflect overall trends, with limited capability to capture in-game state fluctuations, tactical adjustments, and offensive–defensive rhythm changes. With further development, prediction has shifted from pre-match estimation to dynamic modeling during matches. To address the difficulty of real-time outcome prediction, Hawkins et al. (130) developed a volleyball win probability prediction model based on play-by-play data, enabling dynamic estimation of in-set winning probability through continuous updates of match states. By incorporating multidimensional features, including team performance, set scores, and pre-set information, the model updated win probability after each rally, extending prediction from a single time point to the entire course of the match and thereby enhancing its utility for match monitoring and real-time decision support.

**Figure 7:**
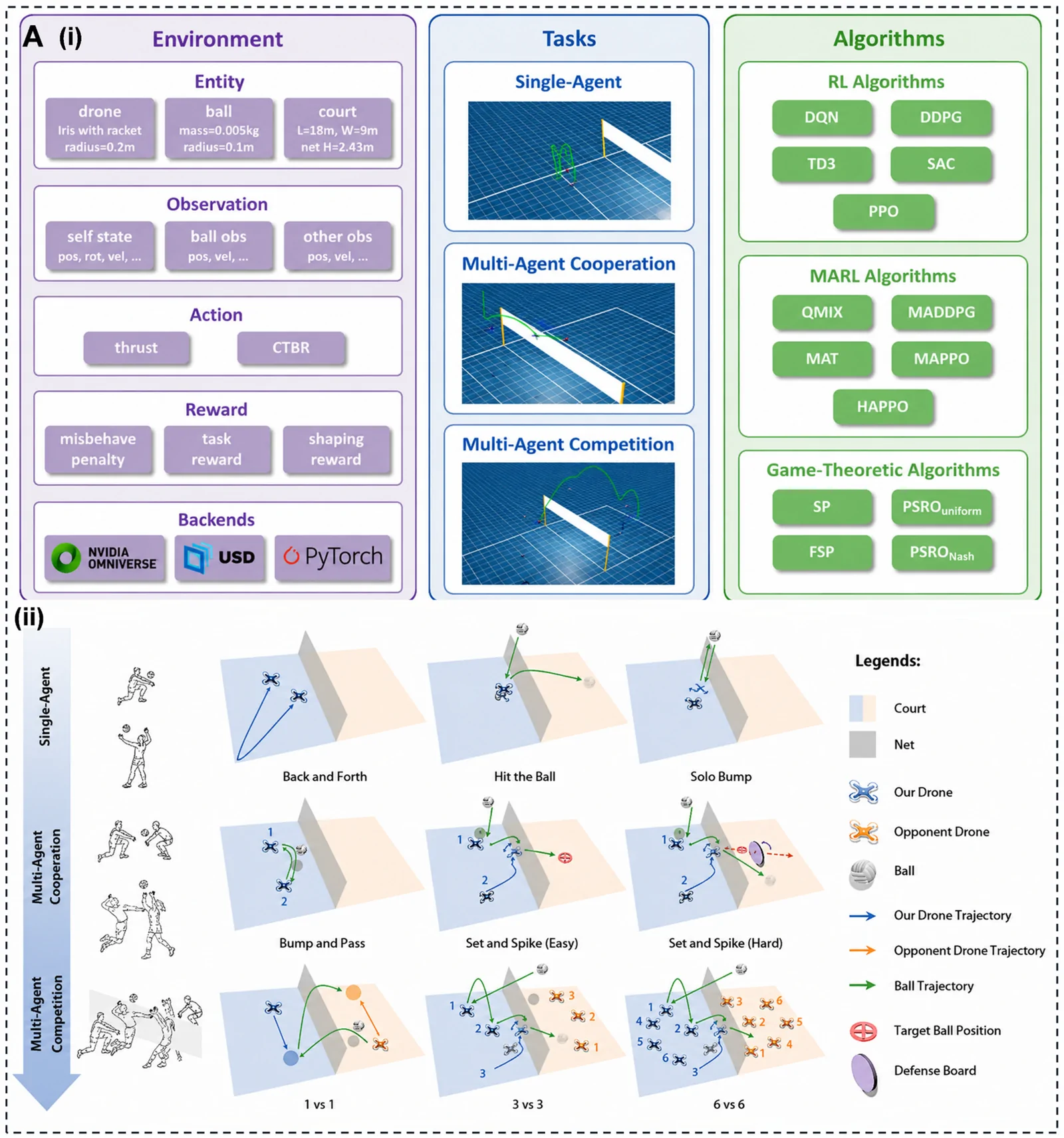
Schematic illustration of volleyball match outcome prediction based on wearable sensing. **(A)** Multi-agent simulation and strategy learning framework based on VolleyBots. (i) Overall architecture of the VolleyBots test platform, including environment modeling (entities, observations, actions, and rewards), task settings (single-agent, multi-agent cooperation and competition), and algorithm modules (reinforcement learning, multi-agent reinforcement learning, and game-theoretic methods); (ii) Progressive task design in the VolleyBots platform, ranging from basic single-agent training to multi-agent cooperative and competitive scenarios, for learning motion control, coordination, and strategic decision-making in volleyball (131).

In addition, Powers et al. (37) attributed changes in win probability to specific technical actions, enabling unified quantitative evaluation of serving, attacking, and defensive performance. This approach provides a common quantitative framework for comparing the contributions of different technical actions to match outcomes and facilitates the identification of key factors influencing match results. However, such methods inherently rely on predefined state transition structures and event definitions; although they quantify contributions, they may not fully capture players’ roles in off-ball movement, tactical positioning, and implicit coordination. Building on this, prediction has further extended to modeling the physical characteristics of actions. Bansal et al. (126) developed an end-to-end deep learning model based on high-speed video data to estimate volleyball spin with high precision. By incorporating physical properties into the predictive framework, the model not only describes what occurs during a match but also provides insight into why certain technical outcomes arise, thereby enhancing the granularity of analysis. However, this approach typically requires high-speed imaging, high-quality video, and accurate object tracking, limiting its applicability in standard match recordings and real training environments. Meanwhile, Xu et al. (131) proposed VolleyBots, a multi-agent simulation framework for volleyball scenarios (Figure 7A), which integrates environment modeling, task design, and policy learning to achieve unified modeling of match processes and decision-making behaviors. The framework defines key elements such as the ball, players, and court, and constructs an interaction mechanism comprising state observations, action spaces, and reward functions. Through a progressive design from single-agent control to multi-agent cooperation and competition, the model learns complex behaviors ranging from basic motion control to coordinated team tactics. By combining reinforcement learning with game-theoretic strategies, the system enables policy optimization in virtual environments and repeated simulation of diverse match scenarios, providing forward-looking support for real-world decision-making.

## 5 Conclusion and perspectives

In recent years, AI and wearable technologies have emerged as key technological drivers for the intelligent development of volleyball analytics. This review systematically summarizes the current advances in AI-based approaches, including machine learning and deep learning, and reviews data acquisition technologies based on IMUs, flexible sensors, and multimodal wearable systems. It further synthesizes their applications in action recognition, event detection, behavior modeling, athlete performance evaluation, training load monitoring, training decision support, tactical optimization, and match outcome prediction. Overall, AI provides intelligent computational capabilities for analyzing complex movement information, whereas wearable technologies offer reliable support for continuous motion sensing. The deep integration of these technologies is accelerating the transition of volleyball analytics from experience-driven to data-driven paradigms and from offline statistical analysis toward real-time intelligent analytics.

More fundamentally, the development of volleyball analytics has shifted from improving the performance of individual models toward the construction of integrated intelligent analytical frameworks. Machine learning, with its strong interpretability, has established a data-driven foundation for match outcome prediction, athlete performance evaluation, and identification of key influencing factors. Deep learning has further advanced the field by addressing challenges associated with object detection, action recognition, multi-agent behavior understanding, and tactical pattern modeling in complex match scenarios, thereby extending volleyball analytics from localized action recognition to team-level coordination analysis and comprehensive match behavior understanding. Meanwhile, wearable technologies have enabled continuous monitoring of training load, technical execution, and physiological status, while their integration with AI algorithms has further facilitated intelligent training feedback, quantitative performance evaluation, and match analysis. Consequently, the convergence of AI and wearable technologies has not only enhanced the automation of sports data acquisition and analysis but also promoted the deep integration of training monitoring, match analytics, and scientific decision-making, providing essential support for the development of intelligent analytical frameworks spanning data acquisition, behavior understanding, sports analytics, and decision support.

Despite the substantial progress achieved in recent years, the large-scale deployment of AI and wearable technologies in real-world volleyball training and competition remains challenging. Most existing AI models still rely on relatively small, labor-intensive annotated datasets, and their generalizability across teams, competitive levels, and match environments requires further improvement. Moreover, challenges associated with multi-player occlusion, rapid offense–defense transitions, and long-term temporal behavior modeling continue to limit model robustness and stability in complex match scenarios. Wearable technologies also face practical constraints, including wearing comfort, long-term operational stability, multi-sensor synchronization, energy supply, and data standardization. In addition, the lack of unified frameworks and evaluation protocols for multimodal heterogeneous data fusion further hinders the widespread adoption of AI-driven volleyball analytics in real-world applications. Future research should place greater emphasis on the development of systematic, intelligent, and application-oriented volleyball analytics. On the one hand, establishing large-scale open-access volleyball datasets, strengthening the integration of multimodal data from vision, inertial sensing, physiological monitoring, and match statistics, and developing lightweight, interpretable, and real-time AI models will be essential for improving generalization capability and deployment efficiency in complex competitive environments. On the other hand, closed-loop intelligent analytical frameworks spanning data acquisition, motion sensing, behavior understanding, training feedback, and tactical decision support should be established to facilitate the translation of AI technologies from laboratory validation to practical deployment in real-world training and competition. Furthermore, emerging technologies, including digital twins, edge intelligence, generative AI, and multi-agent collaborative learning, are expected to further advance volleyball analytics and provide new technological pathways toward more accurate and efficient training monitoring, match analysis, and scientific decision-making.

Overall, this review highlights that the deep integration of AI and wearable technologies is transforming volleyball analytics from conventional experience-driven analysis toward an intelligent analytical framework spanning the entire workflow from data acquisition and motion sensing to behavior understanding, athlete performance evaluation, training optimization, and tactical decision-making. Through the deep coupling of sensing, modeling, analysis, and decision-making, AI is expected to become a key technological bridge connecting athletes, coaches, and the entire competitive process, thereby continuously advancing scientific training, match analysis, and competitive performance in volleyball.
